# S37. STATE-DEPENDENT EFFECTS OF D2 PARTIAL AGONIST ARIPIPRAZOLE ON DOPAMINE NEURON ACTIVITY IN THE MAM NEURODEVELOPMENTAL MODEL OF SCHIZOPHRENIA

**DOI:** 10.1093/schbul/sby018.824

**Published:** 2018-04-01

**Authors:** Susan Sonnenschein, Kathryn Gill, Anthony Grace

**Affiliations:** University of Pittsburgh

## Abstract

**Background:**

Aripiprazole is an antipsychotic drug characterized by partial agonist activity at D2 receptors that impacts both hyperdopaminergic and hypodopaminergic states. It is unclear whether aripiprazole reduces dopamine neuron activity via inhibition or by excitation-induced depolarization block, the latter being characteristic of D2 antagonist administration, and how aripiprazole interacts with D2 antagonist-induced reduction in dopamine neuron activity.

**Methods:**

Adult offspring of saline and MAM-treated rats received aripiprazole (10 mg/kg), or vehicle, p.o. and dopamine neuron activity was examined 2h following acute treatment, or after 1d or 7d withdrawal from 21d repeated treatment. Dopamine neuron activity in the VTA was measured using in vivo extracellular recordings from anesthetized rats. After electrophysiological sampling, apomorphine (200 µg/kg i.p. or 20 µg/kg i.v.) was administered, followed by resampling the VTA to test for the presence of depolarization block. Additional recordings were conducted in MAM rats 1 h following acute haloperidol treatment (0.6 mg/kg, i.p). After electrophysiological sampling, aripiprazole (1mg/kg, i.p.) was administered to examine its effect on haloperidol-induced depolarization block.

**Results:**

Both acute and repeated administration of aripiprazole reversed the increased number of spontaneously active dopamine neurons in MAM rats without impacting control rats. The reduction in dopamine neuron activity persisted after 7d withdrawal from repeated aripiprazole treatment and was not impacted by administration of apomorphine. In contrast, aripiprazole increased dopamine neuron activity in haloperidol-treated MAM rats.

**Discussion:**

This study establishes that aripiprazole rapidly reduces hyperdopaminergic activity in MAM rats, without impacting dopamine neuron population activity in normal rats. The reduction is not due to depolarization block and persists 1 week following withdrawal from repeated treatment. Aripiprazole also removes haloperidol-induced depolarization block in MAM rats, which may underlie the acute psychotic symptoms observed clinically following the switch from D2 antagonist to aripiprazole treatment.

